# Size distributions of fractures, dykes, and eruptions on Etna, Italy: Implications for magma-chamber volume and eruption potential

**DOI:** 10.1038/s41598-019-40563-0

**Published:** 2019-03-11

**Authors:** Salvatore Scudero, Giorgio De Guidi, Agust Gudmundsson

**Affiliations:** 1 Istituto Nazionale di Geofisica e Vulcanologia, Osservatorio Nazionale Terremoti, Via di Vigna Murata 605, 00143 Rome, Italy; 2 Department of Biological, Geological and Environmental Sciences – Earth Sciences Section – University of Catania, Corso Italia, 57, 95129 Catania, Italy; 3 Department of Earth Sciences, Royal Holloway University of London, Egham, TW20 0EX United Kingdom

## Abstract

The main magma source for eruptions on Etna (Italy) is poorly constrained. Here we use data on the size distributions of volcanic fissures/feeder-dykes, crater cones, dyke thicknesses, and lava flows to estimate the average magma volume flowing out of the chamber during eruptions and the volume of the chamber. For the past four centuries the average magma volume leaving the chamber during each eruption is estimated at 0.064 km^3^. From the theory of poroelasticity the estimated chamber volume is then between 69 and 206 km^3^. For comparison, a sill-like, circular chamber (an oblate ellipsoid) 1 km thick and 14 km in diameter would have a volume of about 154 km^3^. The elastic strain energy stored in the host rock during inflation of such a chamber is about 2.8 × 10^14^ J. Estimating the surface energy of a typical dyke-fracture as about 10^7^ J m^−2^, the results suggest that the stored strain energy is sufficient to generate a dyke-fracture with an area of about 28 km^2^. The average strike-dimension of volcanic fissures/feeder-dykes in Etna is about 2.7 km. It follows that the estimated strain energy is sufficient to generate a feeder-dyke with a strike-dimension of 2–3 km and with a dip-dimension as great as 10 km, agreeing with the maximum estimated depth of the magma chamber.

## Introduction

Volcanoes are open thermodynamic systems: they exchange energy and matter with their surroundings. In particular, volcanoes receive heat and magma from their source chambers. Volcanoes also store elastic (mainly strain) energy, both through work done on them by external (e.g. spreading-related) forces and, in particular, through magma-chamber expansion and inflation during unrest periods^1^. The elastic energy is partly transformed into surface energy for the formation of fractures, such as tension fractures, normal faults, and dykes. In order to estimate the elastic energy available to form a feeder-dyke and squeeze magma out of the chamber and to the surface, information on the magma-chamber size is needed.

Here we report for the first time the statistical size distributions of various types of volcanotectonic structures compiled from measurements on a single volcano, namely Etna (Italy), and show how these can be used to estimate its magma-chamber volume and elastic energy during inflation. The features measured include (i) lengths and orientations of volcanic (eruptive) fissures, (ii) thicknesses and orientations of dykes, (iii) diameters, volumes, and orientations of scoria cones, and (iv) combined volumes of feeder-dykes and lava flows. We show that the size distributions generally follow power laws. Using the combined volumes of feeder-dykes and lava flows together with basic poroelasticity theory, we estimate the likely volume of the main magma chamber of Etna. From the chamber volume we estimate the elastic strain energy stored in the volcano during inflation periods. From the estimated strain energy, we infer the potential of dykes injected from the magma chamber to reach the surface so as to supply magma to eruptions in Etna.

### Volcanotectonic setting and activity of Etna

The activity of the Etna volcano, located along the eastern coast of Sicily (Italy), began at ~0.6 Ma^2^; it is presently one of the world’s most active volcanoes. Etna shows all the geochemical features of an ‘anorogenic’ volcano^3^ although its location at the front of the Apennines chain is structurally connected with the subduction and roll-back of the Ionian slab. Theories on the origin of Etna commonly include asthenospheric flow from the descending slab at its lateral edge as a major process^4–7^. At a local scale, the volcano is subject to an ESE-WNW-striking extension, generating a belt of NNW-SSE to NNE-SSW-striking normal fault segments that dissect the lower eastern flank of the volcanic edifice^8^. The volcanic edifice itself rests on a sedimentary basement which reaches the elevation of about 1100 m a.s.l. The entire area has experienced a large crustal-doming episode since 600 ka which has been interpreted as the result of emplacement of altered oceanic crustal material in the deepest parts of the crust^9,10^.

The initial phase of tholeiithic/transitional fissure eruptions (from ~600 to ~320 ka)^11,12^ was followed by the formation of scattered eruptive centres and, finally, the main volcanic axis or zone. The present volcanic edifice has formed along that zone during the past 220 ka and is characterised by Na-alkaline products. Cyclic lateral collapses came in succession, primarily in the period from ~105 to ~15 ka^12^, and the present-day evolution phase of the volcano started 14–15 ka ago. This last phase has been characterised by summit and lateral effusive eruptions, while some plinian eruptions have also been recorded^13^.

One of the main morphological features of the volcanic edifice is a 6.5 × 5 km landslide depression (“Bove” valley) that was generated on the eastern flank in the period from about 8 to 5 ka^14^. In addition, there are hundreds of monogenic vents and scoria (cinder) cones on the slopes of the edifice, mostly located at elevations between 400 and 2800 m a.s.l. The scoria cones and numerous linear features, such as tension fractures and volcanic fissures, show roughly a general radial distribution while some preferential directions can also be recognised^15,16^.

## Results

### Size distributions

**Figure 1:**
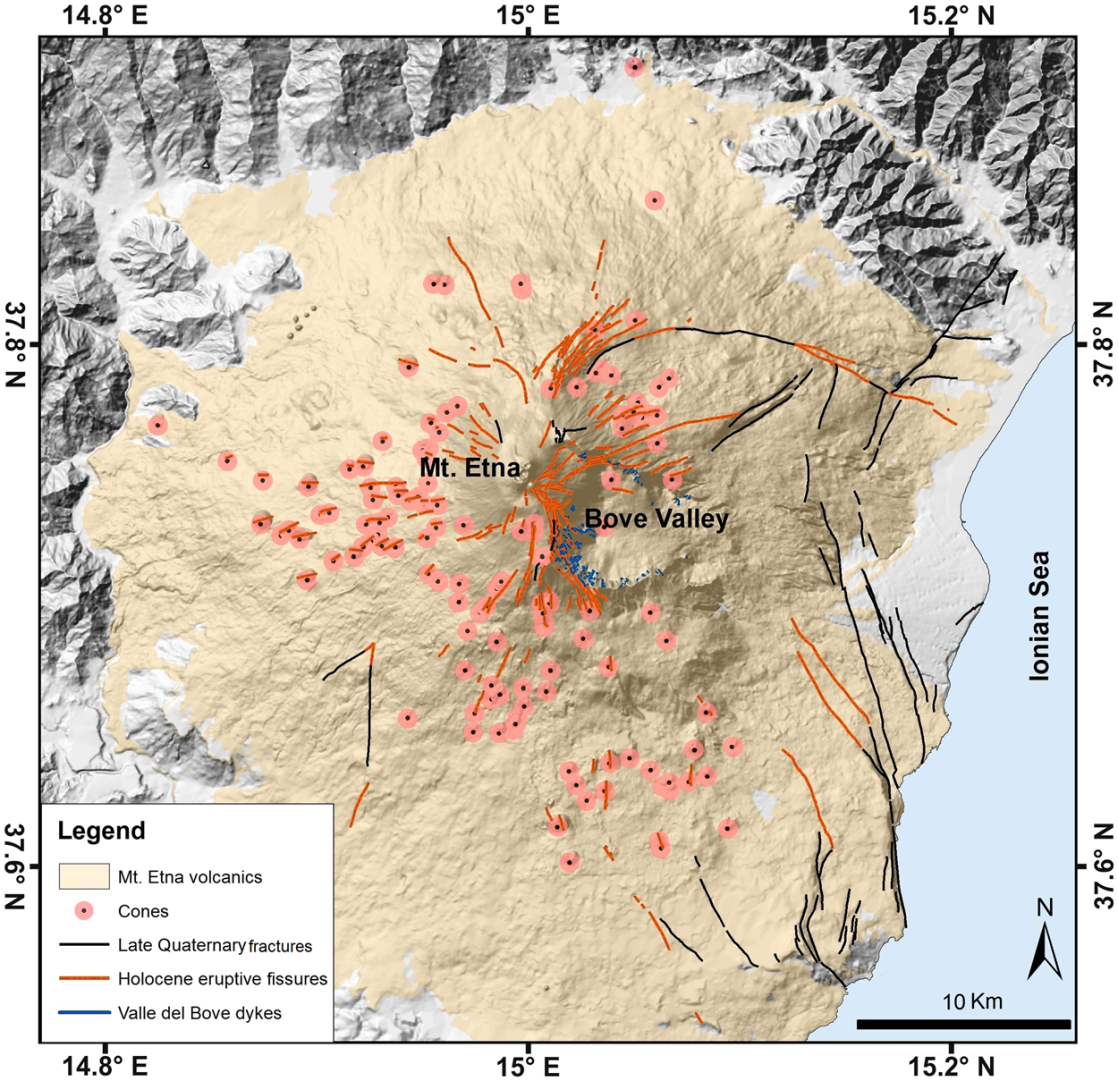
Structural setting of the Etna volcano showing the location of the studied volcanotectonic features (see the text for details). Map composed in ESRI ArcGIS v. 10.2 (http://www.esri.com/arcgis).

**Figure 2:**
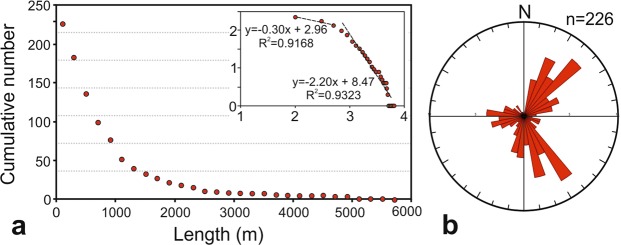
(**a**) Lengths of volcanic fissures plotted as a cumulative frequency size distribution as both a linear and log-log plot (inset) and (**b**) fissures orientation.

**Figure 3:**
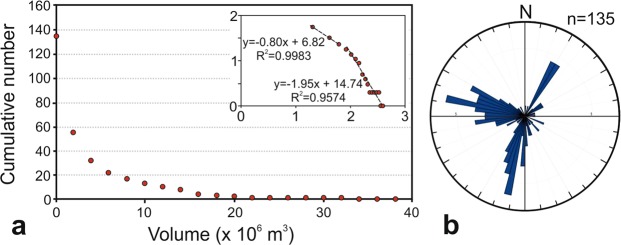
(**a**) Scoria cone volumes plotted as a cumulative frequency-size distribution as both a linear and a log-log plot (inset) and (**b**) the cone azimuthal distribution.

**Figure 4:**
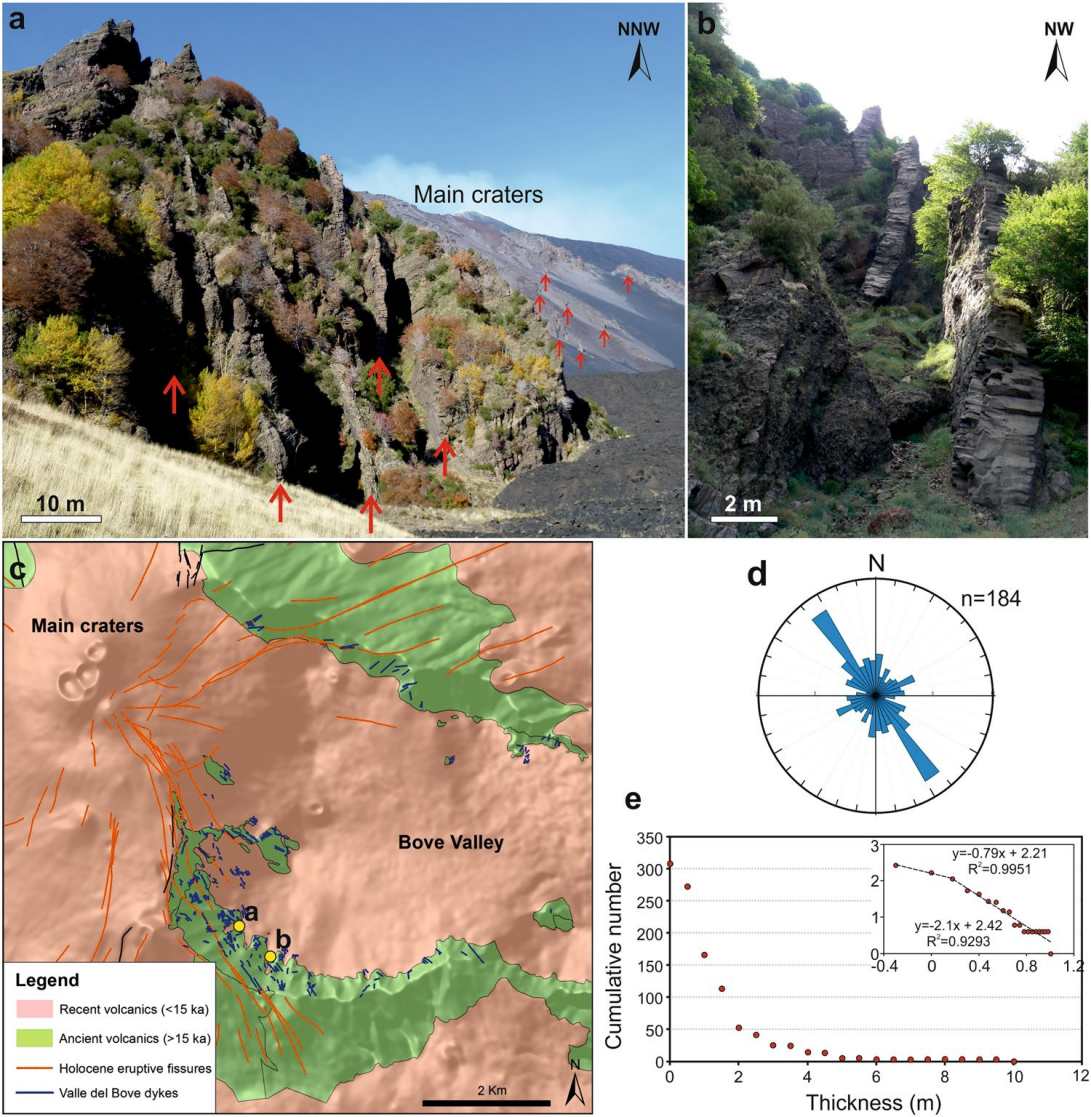
(**a**,**b**) Pictures showing dykes in “Bove” valley. The red arrows indicate some of the dykes. (**c**) A simplified geological map of the valley; 15 ka is the limit of the last eruptive phase of Mt. Etna^12^. The yellow dots indicate the sites where the photographs a and b were taken. (**d**) Orientation (here a symmetric rose) of the dykes. (**e**). Thickness distribution of 184 dykes, plotted as a cumulative frequency distributions as both a linear and a log-log plot (inset). Map composed in ESRI ArcGIS v. 10.2 (http://www.esri.com/arcgis). Photos: Salvatore Scudero.

We analysed three different populations of volcanotectonic structures on Etna (Fig. 1), namely: (a) lengths of eruptive fissures and related tension fractures, (b) volumes of scoria cones, and (c) thicknesses of exposed dykes (excluding late Quaternary fractures), all of which are compiled from published data^16–20^. All these datasets follow, crudely, power-law size distributions when plotted as a cumulative frequency distribution (Figs 2, 3 and 4). When power laws are derived for histograms with certain class limits or bin widths the scaling exponent (the slope of the straight line) depends on chosen bin width or class limits. There is, in addition, commonly considerable noise at the lower end of the straight line (where the size values are larger). This is because at the lower end there are few, and for some bins no, samples or measurement values, so that there are large fluctuations in the counts^21–24^. We therefore use cumulative frequency distributions rather than histograms, whereby one plots the probability *P(x)* that *x* has a value greater than or equal to *x*. The formula is^21–24^:

1 $$P(\ge x)=C{x}^{-D}$$

where *P*(≥ *x*) is the number or frequency of elements with dimensions (here the lengths of fractures, volumes of scoria cones, or thicknesses of dykes) larger than *x*; *C* is a constant of proportionality and *D* is the scaling exponent. A power law can also be presented by taking the logarithms on both sides of the Eq. (1), in which case the equation becomes:

2 $$\mathrm{log}\,P(\,\ge \,x)=\,\mathrm{log}\,C-D\,\mathrm{log}\,x$$

a linear equation. A common procedure for testing if size distributions, such as those above, are really power laws is to log-transform by plotting the data on a bi-logarithmic (log-log) plot. A resulting straight line is then regarded as a general indication that the data follow a power law whose slope is *D*, the scaling exponent (Eq. 2). Because the number of structures or objects decreases as they become larger the slope is negative. The scaling exponent *D*, however, is defined as the negative of the slope and is thus a positive number. To find out if a power law gives the best fit to the data, or if some other functions give a better fit, several different types of tests can be used. The functions commonly considered and compared as regards fit with the power law include log-normal, exponential, and stretched exponential^22,24^. The tests include using the residuals of the curve-fitting procedure, namely the vertical distances of all the points from the regression line. The maximum likelihood method can also be used to compare the power-law fits with log-normal, exponential, and stretched exponential fits. Our results suggest that the size distributions discussed below are well presented by power laws.

### Fracture lengths

The selected fracture population of Etna comprises 226 large-scale volcanic fissures and tension fractures^16–18^. In the lava flows all the tension fractures and volcanic fissures fractures initiate from columnar (cooling) joints^22,25^, formed during solidification and shrinkage of the lavas. These are with dimensions of the order of tens of centimetres to metres. All the selected fractures discussed here have dimension greater than 100 metres.

Volcanic fissures are fluid-driven fractures (hydrofractures) which, together with tension fractures, are extension fractures or mode I cracks^25^. The fracture-forming extension is partly driven by magmatic pressure in dykes, partly by volcano spreading. Dykes that reach within metres or tens of metres of the surface, many eventually becoming feeders, commonly trigger the formation or reactivation of tension fractures and small normal faults^26^. In Etna and other large edifices away from divergent plate boundaries, a combination of volcano spreading and dyke intrusion is one main reason for the formation of tension fractures and small normal faults. The tensile stresses are then partly related to magmatic overpressure and partly to spreading and become concentrated in the layers above the arrested dyke, which then acts as a narrow notch or edge crack^25^. Extension-fracture opening and propagation events on Etna take from hours to weeks and are normally accompanied by migrating earthquake swarms^26^. Explosive or effusive features such as vents, scoria cones, pit craters, and hornitos are often aligned along these fractures.

The length-size distribution of the 226 fractures follows a power law. This is indicated by the ordinary plot in Fig. 2a, and also seen on the log-log plot on the inset. In fact, the inset shows that the length-size distribution follows strictly two straight lines, that is, power laws with different scaling exponents – double power laws. Such a break in a power law is common for fractures and normally indicates a ‘phase change’; in the present context a change in the mechanics of fracture formation^22,25^ and/or changes in the host-rock properties. In the present case, the break may be partly related to the tension fractures tending to be shorter, and with a lower scaling exponent, than the volcanic fissures. This can be understood in terms of a change in the mechanics of fracture formation. The tension fractures form in response to absolute tensile stresses related to rifting or, for Etna, more specifically volcano spreading. By contrast, the volcanic fissures are primarily driven open by internal magmatic pressure, namely overpressure (or driving pressure), which is commonly considerably higher than the tensile stress forming tension fractures^25^.

The azimuth direction of each measured fracture is taken from the tip located at the higher altitude (upslope) to the one at a lower altitude (downslope). The data set is thus directional. Three main strike peaks are clearly recognisable, namely at ~40°, at ~150°, and at ~270° (Fig. 2b). Each of the three clusters represented by these peaks spans some 60–70°. The first subset (10–80) constitutes 31% of the cumulative fracture length and 34% of the total number of fractures, the second subset (140–200°) 33% of the length and 31% of the number, and the third subset (230–300°) 15% of the length and the 20% of the number. The first and second subsets are oblique to the local spreading or extension vector in the edifice, striking ~100° and largely controlled by the regional tectonics in the area^8^, whereas the third subset is almost parallel to the vector. Those three sets or clusters coincide with the three main rift zones active in Etna during the past 15 ka^15^.

### Scoria-cone volumes

The main eruptive vents are located in the summit area, above about 2900 m a.s.l., but hundreds of monogenetic scoria cones that have formed during flank eruptions are scattered all around the flanks of the volcanic edifice. Their spatial distribution is partly controlled by the occurrence of three radial rift zones^15,27^: the NE, S and W rift zones (Fig. 3b). As for the volcanic fissures, the temporal span of the cone formation is limited to the last eruptive phase of Etna. The most complete catalogue of scoria cones on Etna was compiled by Favalli *et al*.^19^ and contains the geographic references and several geometrical parameters (e.g. diameters, heights, slope, volume, etc.) for 135 scoria cones. The azimuthal strike distribution of fractures shows two main peaks at about 30–50° and 150° and a secondary peak at about 270°; for the scoria cones there are three main peaks centred at about 30°, 190–200°, and 280–290° (Figs 2b, 3b). There is thus a partial overlap in the peak strike distributions of volcanic fissures and scoria cones with that of the main rift zones.

We tested the power-law distribution for the diameter, height, and volume of the scoria cones, the average values for these parameters being 390 m, 38 m, and 3.6 × 10^6^ m^3^, respectively. All the parameters show power-law fits. As an example, in Fig. 3a we show the volume-size distribution of the scoria cones.

### Dyke thicknesses

The “Bove” valley (Fig. 4a,b) is a horseshoe-shaped collapsed sector on the seaward flank of the volcanic edifice, the result of the combination of morphological and volcanotectonic processes^14^. On the steep margins of the depression many dykes and sills are exposed (Fig. 4a). Studies of the dykes include petrographic^28^ and stress-field analyses^29,30^ as well as analyses of magma propagation directions^31^. Following these, Ferrari *et al*.^20^ measured orientation and thickness of 184 dykes. The thickness ranges between 0.2 and 10 m, the average thickness being 1.84 m. Because the dykes outcrop on the deeply eroded margins of the valley, the original landforms are not preserved and it is commonly not possible to establish whether dykes were feeders or non-feeders. Dykes are predominantly vertical (Fig. 4a) or sub-vertical (they dip 84° on average) and follow a dominant NW-SE trend (Fig. 4c); again, we successfully tested and found a power-law fit for the dyke-thickness dataset (Fig. 4d).

Knowing the dimensions of a dyke, it is possible to calculate the volume of magma needed to form it. Since it is not possible to measure the lateral extensions of dykes in the field in Etna, we use a typical shallow-depth (crustal depths less than about 1 km) dyke length/thickness ratio of 1500^25,32^ giving an average length (i.e. strike dimension) of 2760 m. This ratio takes into account the increasing lateral or strike dimension (‘lengths’) of dykes in relation to dyke thickness with increasing crustal depth as a result of increasing Young’s modulus^25^. Many dykes inject and propagate from the roof of the source magma chamber, whose depth is at about 3–6 km b.s.l, or about 6–9 km below the summit of Etna, based on petrochemical, geophysical, and geodetic data^33–39^. As discussed below, other dykes, however, are injected laterally from the central conduit of Etna.

Giving the dyke dimensions considered above, we can calculate the volume of magma that must flow out of the chamber to form a dyke of such dimensions. Taking into account the uncertainty of the depth of the magma chamber, that is, the dip dimensions of the dykes, the common dyke volume is estimated as between 2.5 × 10^7^ m^3^ and 4.1 × 10^7^ m^3^.

### Eruption volumes

The volume of magma (and other fluids) that flows out of a magma chamber during rupture and dyke injection, often resulting in an eruption, depends on the size and the mechanical properties of the chamber in relation to those of the host rock. Using a poroelastic model^32,40^, we here use the outflowing magma volume, based on calculated dyke and eruption volumes, to estimate the volume of the Etna chamber.

There are several datasets on the volumes of eruptions in Etna^41,42^. The average eruptive volumes in the datasets are similar, the average volume of the individual lava flows being 6.25 × 10^6^ m^3^.

When considering the recurrence time of the eruptions, the calculated average eruptive volumes are also consistent with the average eruptive rate of Etna in the past 60 ka, estimated at 4.8 × 10^6^ m^3^/a^43^. Most volumes are estimated from the shapes and sizes of the lava fields and associated scoria cones and pyroclastic products and thus refer only to the eruptive materials. In the past few decades, however, GPS and interferometric techniques allow dyke and other intrusion volumes, as well as the magma-chamber inflations, to be estimated approximately from the associated surface deformation^33,44–47^. The average estimated intrusive volume is in the order of 10^7^ m^3^, although bigger intrusions up to 3 km^3^ have been suggested^48^. The average combined extrusive and intrusive (feeding-dyke) volume is thus about 6.4 × 10^7^ m^3^, a value which can be used to estimate crudely of the volume of Etna’s magma chamber.

### Magma chamber volume and strain energy

For a totally molten magma chamber, the chamber volume *V*_*c*_ is related to the average volume of magma *V*_*er*_ transported or flowing out of the chamber through a feeder-dyke (including the volume of the feeder itself) during an eruption via the equation^40,49^:

3 $${V}_{c}=\frac{{V}_{er}}{{p}_{e}({\beta }_{r}+{\beta }_{m})}$$

where *p*_*e*_ denotes the magma excess pressure in the chamber at the time of rupture and feeder-dyke initiation, *β*_*r*_ the host-rock compressibility, and *β*_*m*_ the magma compressibility. The static compressibility *β*_*m*_ for basaltic magma at temperatures of 1100–1300 °C is about 1.25 × 10^−10^ Pa^−1 ^^40,49^, whereas the static host-rock compressibility *β*_*r*_ is estimated at about 3 × 10^−11^ Pa^−1 ^^40,49^. These estimates of the static *β*_*r*_ use the dynamic/static ratio of Young’s modulus of 2^21^. The excess pressure *p*_*e*_ is generally similar to the *in-situ* tensile strength of the roof of the chamber^21,47^. Measured *in-situ* tensile strengths range from 0.5 MPa to 9 MPa, but are most commonly in the range of 2–6 MPa, with an average close to 4 MPa^25,49^. Using the above average extrusive plus intrusive volume and the compressibility values, from Eq. (3) *V*_*c*_ varies from about 206 km^3^ (for *p*_*e*_ = *T*_0_ = 2 MPa) to 69 km^3^ (for *p*_*e*_ = *T*_0_ = 6 MPa), with an average of about 103 km^3^ (for *p*_*e*_ = *T*_0_ = 4 MPa).

These are very reasonable values and are all within commonly estimated magma-chamber sizes. For example, a sill-like circular chamber with a radius of about 4.7 km and a thickness of 1 km would have the minimum estimated volume of 69 km^3^. Similarly, a circular sill-like chamber with a radius of 5.7 km and a thickness of 1 km would have the average estimated volume of 103 km^3^. For the largest volume, a circular sill-like chamber, again 1 km thick and with a radius of 8 km would have the volume of 206 km^3^. Given the size of Etna, a chamber with a diameter of 9–16 km is very plausible. Sill-like (oblate ellipsoid) geometry appears to be the most common among magma chambers worldwide^1,49,50^. In the absence of evidence to the contrary, it is thus reasonable to assume that shape for the Etna magma chamber. As indicated above, petrological and geophysical data suggest a depth for Etna’s chamber of 6–9 km below the top of the volcanic edifice^33–39^.

For a sill-like magma chamber the strain energy *U*_0_ in terms of magmatic excess pressure *p*_*e*_ is given by^1^:

4 $${U}_{0}=\frac{8(1-{\nu }^{2}){{p}_{e}}^{2}{a}^{3}}{3E}$$

where *a* is the lateral radius of the chamber, and *ν* and *E* are the rock Poisson’s ratio and Young’s modulus, respectively. Eq. (4) allows us to calculate the strain energy stored in the edifice of Etna and associated crustal segment for a given chamber excess pressure. The stored strain energy is then partly available for the formation of dyke fractures, as well as tension fractures and normal faults, and, in case of an eruption, for squeezing magma out of the chamber and to the surface^1,49^. For a typical feeder-dyke in Etna and elsewhere the surface energy needed to form the dyke-fracture (and other tectonic extension fractures in rocks) is of the order of 10^7^ J m^−2 ^^1^. Thus, to rupture one square metre of rock during the propagation of an extension fracture such as a dyke, elastic energy of the order of 10^7^ J must be transformed into surface energy, which is the energy needed to rupture the rock and move the rupture surfaces apart^25^.

Using the values discussed above, namely a typical overpressure of 4 MPa, magma-chamber radius of 7 km, a static Poisson’s ratio of 0.25, and a static Young’s modulus of 50 GPa – a reasonable Young’s modulus given the estimated chamber depth (i.e. 3–6 km b.s.l, or 6–9 km below the Etna edifice^33–39^) - Eq. (4) yields strain energy of about 2.8 × 10^14^ J. Thus, the strain energy stored in Etna and the hosting crustal segment due to its magma chamber expansion as a result of overpressure of 4 MPa would be sufficient to generate a dyke-fracture (or other extension fracture) with an area of about 28 km^2^. For example, for the average dyke strike dimension of 2760 m, estimated above, the, for a vertical dyke, the dyke dip dimension, or the depth to the shallow chamber below the top of Etna, could be as much as about 10 km. This is greater than the depth normally assumed for the Etna chamber (6–9 km), indicating that the strain energy is more than sufficient to propagate typical feeder-dykes vertically from a magma chamber of the estimated volume and depth. That the strain energy is theoretically sufficient to produce a feeder-dyke does not mean that an injected dyke necessarily becomes a feeder, however. The paths of dykes depends on many mechanical factors and many dykes become arrested, primarily because of unfavourable mechanical layering and local stresses, and thus never reach the surface to erupt^32,50–53^.

As indicated above, many dykes in Etna form in lateral propagation from the central conduit rather than vertically from the magma chamber. This seems to have been a particularly common mode of dyke emplacement in the volcano during the past century^54^, but over longer periods of time Etna, like other volcanic edifices, is supplied with magma through both laterally and vertically propagating dykes^55–57^. In particular, vertically propagating dykes are needed to form any major volcanic edifice in the first place and then to maintain its geometry. However, the strain-energy model applies also to dykes emplaced laterally from a central conduit or chamber. While we here use the example of vertical dyke paths, the surface energy for any dyke path – vertical, vertical and then lateral, lateral and then vertical, or purely lateral – can be calculated and compared with the available strain energy. The only requirement is that sufficient strain energy accumulates in the volcano (primarily through inflation) before dyke propagation and, eventual, eruption. And there is plenty of evidence for inflation (accumulation of strain energy) prior to dyke injections and eruptions in Etna^44,46,48,58,59^.

**Figure 5:**
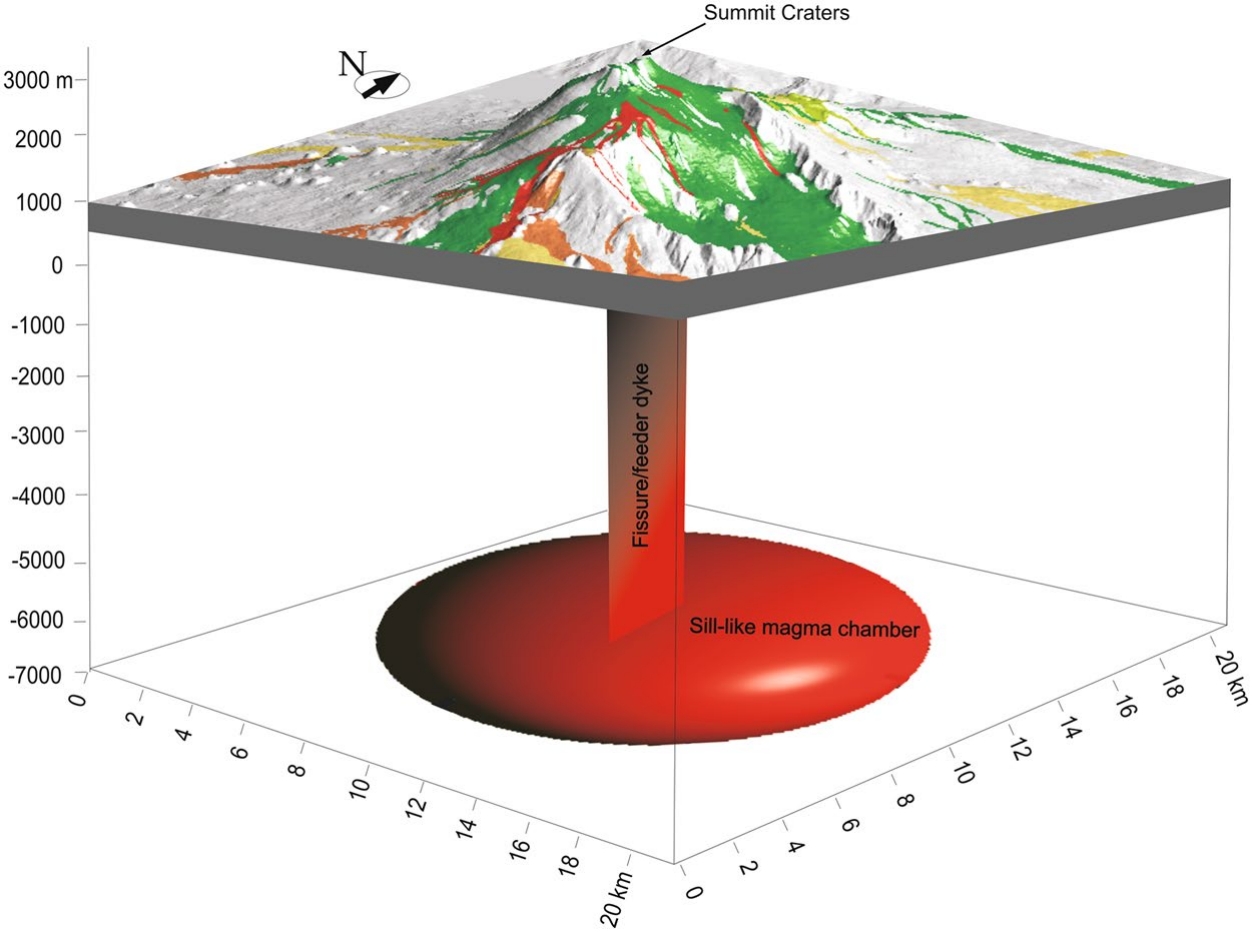
Schematic block diagram showing a 3D view from the south-east of the proposed model: a vertically propagating dyke from the magma-chamber supplies magma to an eruptive fissure at surface. The coloured surfaces represent the historical lava flows dated from 1381 to recent. Map composed in ESRI ArcGIS v. 10.2 (http://www.esri.com/arcgis).

Here we used the chamber radius of 7 km, whereas, based on our chamber volume estimates and assumed thickness of 1 km, the radius should be somewhere between 4.7 and 8.1 km (Fig. 5). If the minimum or maximum radius, 4.7 km and 8.1 km, were used, the calculated strain energy, other things being equal, would be proportionally smaller (8.5 × 10^13^ J for 4.7 km radius) or larger (4.3 × 10^14^ J for 8.1 km radius) than the above value. In all cases, however, the energy would be sufficient to generate dykes and other extension fractures with dimensions of the order of kilometres or larger. There may also be additional elastic energy stored in the volcanic edifice of Etna due to its spreading^1^. During feeder-dyke formation and eruption, part of the strain energy is used to squeeze out the magma from the chamber^47^ and for the formation and development of normal faults and tension fractures. The main result from these calculations, however, is that for the estimated size and properties of the Etna magma chamber, a typical excess pressure before its rupture and dyke injection generates elastic strain energy that is, theoretically, sufficient for a vertically propagating dyke to reach the surface.

That many of the injected dykes do not, eventually, reach the surface to erupt is thus generally not the result of lack of strain energy that needs to be supplied so that the dyke fracture (the dyke path) can reach the surface. By contrast, dyke arrest in Etna is primarily because of the mechanically contrasting layers that constitute the volcano – as is, indeed, observed in many other volcanoes^30,48,51^. If the typical strain energy generated during unrest periods were less than needed to propagate a typical dyke to the surface of Etna, and particularly the summit part, then gradually the geometry of the volcano would change. Based on the present model that is not the case. Thus, each unrest period with a dyke injection has the potential of giving rise to an eruption in Etna. Those dykes that fail to erupt, become arrested, do so primarily because of unfavourable mechanical layering, and associated local stresses. Understanding the effect of layering on dyke propagation paths, including dyke arrest and lateral versus vertical dyke propagation, is thus of fundamental importance, worldwide and, in particular, for Etna.

## Conclusions

- Size distributions of various volcanotectonic structures on Etna all follow roughly power laws. Those studied here include (i) lengths of eruptive fissures and tension fractures, (ii) the diameters, heights, and volumes of scoria cones, and (iii) dyke thicknesses. From these data the average length of volcanic fissures/feeder dykes is estimated at about 2.7 km. Using the estimated common dyke dimensions and eruptive volumes during the past several centuries, we estimate a typical combined intrusive (dyke) and extrusive magma volume flowing out of the Etna main source chamber during eruptions as about 0.064 km^3^ (6.4 × 10^7^ m^3^).
- Using this estimate, as well as poroelasticity theory and the *in-situ* tensile strength of the host rock, the volume of the Etna magma chamber is estimated as being in the range of 69–206 km^3^ (6.9–2.06 × 10^11^ m^3^), with a most likely value of 103 km^3^ (1.03 × 10^11^ m^3^). For a sill-like (oblate ellipsoidal) magma chamber with a thickness of 1 km, the lateral radius of the chamber would be from 4.7 to 8.1 km.
- Using a typical magmatic excess pressure of 4 MPa and appropriate elastic constants, a magma chamber of the above size generates an elastic strain energy (through expansion or inflation) in the edifice of Etna and the associated crustal segment of about 2.8 × 10^14^ J. This energy is theoretically sufficient for the formation of a dyke-fracture (or other extension fractures) with an area of about 28 km^2^. The strain energy is thus large enough to generate typical feeder-dyke with a strike-dimension of 2.7 km and height of about 10 km. This latter value is similar the maximum estimated depth of the magma chamber beneath Etna and shows that the typical strain energy stored during inflation is theoretically high enough to propagate a dyke to the surface from the likely depths of the main source magma chamber of Etna.
- Theoretically, the strain energy generated during a typical unrest period in Etna is great enough for an injected dyke to reach the surface. Thus, each unrest with a dyke injection is also a potential eruption. That many of the injected dykes do not, eventually, reach the surface to erupt but rather become arrested is primarily because of the mechanically contrasting layers that constitute the edifice of Etna.

## Methods

The datasets have been compiled through merging and re-sampling various maps at different scales^16–18^. All the fissures relate to the last eruptive phase of the volcano and are therefore younger than 15 ka. We exclude fractures shorter than 100 m because their lengths cannot be measured with sufficient precision on map at scales much smaller than 1;10,000. Also, 100 m is less than the selected bin length (200 m) and negligible considering the average length and the size range of the whole fracture population. The resulting power-law distribution is thus not much affected by this type of censoring^60^. Measured fracture length ranges from 100 to 5600 m, the average length being 887 m and the cumulative total length of the fracture/fissure network about 200 km. The fracture network is located within an area of about 650 km^2^, whereas the total area covered by the volcanic edifice of Etna is about 1260 km^2^.

In Eq. (3) the chamber is assumed totally molten, a standard assumption for inversion of geodetic data to infer the depths to magma chambers. However, many chambers contain a crystal mush and solidified matrix and may therefore be closer to a classic poroelastic material, in which case Eq. (2) becomes modified to:

5 $${V}_{c}=\frac{{V}_{er}}{\eta {p}_{e}({\beta }_{m}+{\beta }_{p})}$$

where *β*_*p*_ is the pore compressibility of the chamber, i.e. the fractional change in pore volume (magma fraction) of the chamber for unit change in the excess pressure, *η* is the porosity, and the other symbols are as defined in Eq. (3). Here new magma received by the chamber is partly accommodated through compression of the old magma in the chamber and partly by expanding the chamber pore space. Generally, *β*_*m*_ is much larger than either *β*_*r*_ (Eq. 3) or *β*_*p*_ (Eq. 5) so that, as regards compressibility, *V*_*c*_ depends primarily on *β*_*m*_, which is calculated using data provided by Murase and McBirney^61^.
